# Research on the Evolution Characteristics and Influencing Factors of Foamy Oil Bubbles in Porous Media

**DOI:** 10.3390/molecules30051163

**Published:** 2025-03-05

**Authors:** Moxi Zhang, Xinglong Chen, Weifeng Lyu

**Affiliations:** 1 University of Chinese Academy of Sciences, Beijing 100049, China; zhangmoxi21@mails.ucas.ac.cn; 2 Institute of Porous Flow and Fluid Mechanics, Chinese Academy of Sciences, Langfang 065007, China; 3 Research Institute of Petroleum Exploration & Development, PetroChina, Beijing 100083, China; chxlhdpu@petrochina.com.cn; 4 State Key Laboratory of Enhanced Oil and Gas Recovery, Beijing 100083, China

**Keywords:** foamy oil, microscopic glass model, bubble growth rate, molecular simulation, VOF

## Abstract

This study systematically investigates the formation mechanism and development characteristics of the “foamy oil” phenomenon during pressure depletion development of high-viscosity crude oil through a combination of physical experiments and numerical simulations. Using Venezuelan foamy oil as the research subject, an innovative heterogeneous pore-etched glass model was constructed to simulate the pressure depletion process, revealing for the first time that bubble growth predominantly occurs during the migration stage. Experimental results demonstrate that heavy components significantly delay degassing by stabilizing gas–liquid interfaces, while the continuous gas–liquid diffusion effect explains the unique development characteristics of foamy oil—high oil recovery and delayed phase transition—from a microscopic perspective. A multi-scale coupling analysis method was established: molecular-scale simulations were employed to model component diffusion behavior. By improving the traditional Volume of Fluid (VOF) method and introducing diffusion coefficients, a synergistic model integrating a single momentum equation and fluid volume fraction was developed to quantitatively characterize the dynamic evolution of bubbles. Simulation results indicate significant differences in dominant controlling factors: oil phase viscosity has the greatest influence (accounting for ~50%), followed by gas component content (~35%), and interfacial tension the least (~15%). Based on multi-factor coupling analysis, an empirical formula for bubble growth incorporating diffusion coefficients was proposed, elucidating the intrinsic mechanism by which heavy components induce unique development effects through interfacial stabilization, viscous inhibition, and dynamic diffusion. This research breaks through the limitations of traditional production dynamic analysis, establishing a theoretical model for foamy oil development from the perspective of molecular-phase behavior combined with flow characteristics. It not only provides a rational explanation for the “high oil production, low gas production” phenomenon but also offers theoretical support for optimizing extraction processes (e.g., gas component regulation, viscosity control) through quantified parameter weightings. The findings hold significant scientific value for advancing heavy oil recovery theory and guiding efficient foamy oil development. Future work will extend to studying multiphase flow coupling mechanisms in porous media, laying a theoretical foundation for intelligent control technology development.

## 1. Introduction

Heavy oil accounts for a huge proportion in the world’s petroleum resources, approximately 50% of the total hydrocarbon reserves [1]. The main characteristic of conventional heavy oil is its high viscosity. However, some oil reservoirs exhibit abnormal production performance during the development process [2,3]. Foam oil is a type of oil with high viscosity, characterized by the presence of bubbles formed due to the release of dissolved gas [4,5].

Foamy oil is essentially different from the mixture of oil and gas two-phase fluids in conventional oil reservoirs, and the corresponding theories and development technologies are also quite abundant [6,7]. Due to the high reserves of heavy oil in Canada and Venezuela and the obvious characteristics of foamy oil, relevant research was carried out relatively early, covering the physical property characteristics of foamy oil, generation and storage conditions, influencing factors for stability, development mechanism and methods for enhancing oil recovery, etc. [8,9]. In recent years, with the development of domestic complex oil reservoirs and the gradual expansion of overseas oilfield development business, certain achievements have also been made in the theoretical understanding and mechanism research of foamy oil [10,11]. The formation of bubbles in foamy oil is a continuous process. The supersaturation of gas components is the fundamental cause of bubble generation [12,13], and the degree of supersaturation determines the sensitivity to factors such as temperature and pressure conditions [14,15,16]. The main factors for the formation (bubble formation) of foamy oil include asphaltene content [17], supersaturated state [18,19], and pressure reduction rate [20,21,22].

The phase change process of high-viscosity crude oil is also the process of the initial generation of bubbles within high-viscosity crude oil (the formation of foamy oil) and the development of bubbles. Generally speaking, the generation of foamy oil mainly consists of the following stages: the initial supersaturated state, bubble nucleation, bubble growth, bubble migration, coalescence, and rupture. According to Alshmakhy’s [23] research, the formation of microbubbles starts at the critical supersaturated pressure (this pressure point is lower than the bubble point pressure of heavy oil), and there is a supersaturation threshold. Only when the gas saturation is greater than this value will bubbles nucleate. Due to the randomness of bubble nucleation, the critical supersaturation is not a definite value but depends on the time of nucleation. The supersaturation process is affected by the pressure depletion rate [24]. Under a high-pressure depletion rate, the increase in supersaturation can shorten the time required for bubble nucleation. As the pressure depletes, bubble nuclei embryos on the molecular scale will be generated inside the liquid and gradually transform into bubble nuclei and microbubbles. Bubble nucleation can be classified into instantaneous nucleation and progressive nucleation according to the nucleation speed [25], and can also be divided into primary nucleation and secondary nucleation according to the nucleation process. Among them, primary nucleation includes homogeneous nucleation and heterogeneous nucleation [26]. Bubble growth is the main process that changes the seepage characteristics of foamy oil and is controlled by two factors: the diffusive effect in the early stage and the pressure-driven effect in the later stage. In the early stage, bubble formation and the migration of bubbles account for the major part, and the capillary force acts as a driving force in the movement process of bubbles. In the later stage, due to the far-reaching influence of pressure depletion, the bubble growth process is related to the pressure depletion rate and the volume expansion of the fluid. In addition, viscous force and capillary force, bubble mass and momentum, heat and mass transfer, interfacial tension, diffusivity and initial bubble radius, crude oil viscosity, components and quantity of dissolved gas in crude oil, temperature and pressure depletion rate also have certain influences on the bubble growth process [27,28]. Under normal circumstances, the migration and growth of bubbles occur simultaneously. Adjacent bubbles are affected by rock particles or other bubbles during their movement. After adjacent bubbles approach each other, the liquid film between them begins to thin slowly and eventually ruptures, and the adjacent bubbles merge into one [29]. Its influencing factors include dissolved gas, pressure depletion rate and flow rate, pore structure and sand production, crude oil components and viscosity, small bubbles and interfacial tension, etc. [30,31,32].

Regarding the microscopic phase behavior of foamy oil, in addition to the research on experimental mechanisms [7,8,10,33,34], the research on mathematical models has also attracted increasingly extensive attention. It has been found through experiments that the formation and coalescence processes of bubbles in foamy oil have a power exponential function relationship with pressure and time. The model of the bubble growth index was adopted to characterize and describe the bubble growth process [26]. Ben Hamida et al. [35] proposed equations for controlling the state of bubbles. Sheng et al. [36] and Joseph et al. [37] both established kinetic mechanism models describing the physical changes of gas dispersed in oil. Shen [38,39] put forward a general dynamic model based on the understanding of the escape process of dissolved gas and a gas lubrication model based on viscosity. Smith [13] established a dispersed microbubble model and used the mixture characteristic equation to describe the flow of oil–gas mixtures. Gennady et al. [40] put forward the concept of pseudo-bubble point pressure and described the depletion production of heavy oil reservoirs. The established pseudo-bubble point model can calculate the properties of conventional experimental fluids, but it is difficult to simulate the time-related changes in the flow characteristics of foamy oil.

The growth status of bubbles is the key in the “foamy oil flow” stage. Based on the current research status of the above experiments and numerical simulations, there is currently a lack of analytical models for studying the influencing factors of bubble growth from the perspective of physical chemistry. In this paper, according to the differences in physical property parameters between foamy oil and ordinary heavy oil, the influence degrees of crude oil components, viscosity, and interfacial tension on bubble growth are analyzed. By combining the microscopic experiment on the pressure drop of foamy oil with molecular simulation and the numerical simulation technology of the VOF (Volume of Fluid) method [41], a quantitative analysis is carried out on the main influencing stages and the contribution degrees of the effects of these three factors, and an empirical formula for bubble growth is obtained. Through the empirical formula, a deeper understanding of the phase change process of foamy oil and the seepage characteristics of bubbles in porous media is achieved, which provides theoretical guidance for the depletion production time of foamy oil and the application of subsequent production technologies.

## 2. Methods

### 2.1. Experimental Samples

Foamy oil mainly includes the following stages: the initial supersaturated state, bubble nucleation, bubble growth, bubble migration, coalescence, and rupture. This paper only focuses on the growth process of bubbles and its influencing factors. The microscopic etched glass model experiment is an effective means to observe the microscopic phase change characteristics of foamy oil in porous media. Therefore, it is used to conduct the foamy oil pressure drop experiment. With the aim of observing the bubble growth process, a microscopic etched model with the characteristics of the pores of real oil reservoir rocks is selected to conduct an in-depth study on the growth characteristics and influencing factors of bubbles in foamy oil.

**Figure 1:**
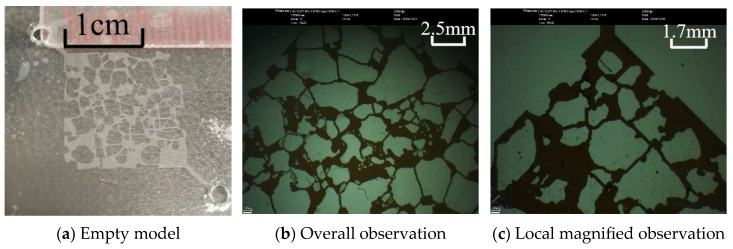
Microscopic glass etched model.

Figure 1 illustrates the appearance and dimensions of the microscopic glass etched model. Figure 1a shows the blank model, with an effective area of 15 mm × 15 mm and an etched depth of 20 μm. The injection and production ends are located in the diagonal direction. Figure 1b presents the pore image after being saturated with oil, and as shown in the magnified view in Figure 1c, the minimum pore diameter is 75 μm.

The experiment selected foam oil from the Orinoco region of Venezuela as the research subject. The formation oil has a viscosity of 5873 mPa·s, while the degassed crude oil has a viscosity of 50 mPa·s. To avoid pore blockage by heavy components during the experiment, a flowable simulated oil was prepared according to the component ratio for the study. The oil sample and natural gas were collected from the field in Venezuela and consist of degassed crude oil and natural gas samples. The simulated oil and natural gas were mixed at a constant pressure to prepare the simulated oil sample, with an oil-to-gas ratio of 7.1 m^3^/m^3^, resulting in a saturated foam oil viscosity of 12 mPa·s (@50 °C, 3.0 MPa). The viscosity of the oil sample was measured using a Brookfield DV-II+Pro rotational viscometer (Toronto, ON, Canada) under experimental temperature and pressure conditions, employing the equilibrium method. The components of the degassed crude oil and natural gas are listed in Table 1 and Table 2, respectively.

### 2.2. Experimental Setups

**Figure 2:**
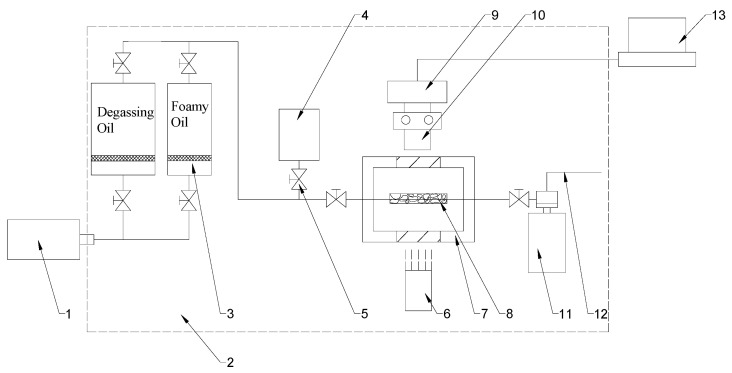
Flow chart of the microscopic experiment on foamy oil pressure drop. 1. Micro-pump; 2. Constant temperature box; 3. Intermediate container; 4. Vacuum pump; 5. Valves; 6. Light source; 7. Visible high-pressure model; 8. Glass model; 9. High-speed camera; 10. Microscope; 11. Back pressure valve; 12. Export; 13. Image acquisition and display system.

The apparatus for the foamy oil pressure-drop experiment uses a microscopic glass etched model, and the flow chart is shown in Figure 2. The experimental apparatus mainly includes: a micro-pump (Quzix-5210, Chandler, AZ, USA, with a minimum controllable speed of 0.00001 mL/min and a maximum speed of 10.0 mL/min), a visual high-pressure model (with a maximum pressure resistance of 20 MPa and a temperature of 90 °C), a glass model (with pores ranging from 100 to 1000 μm), a microscope (Carl Zeiss Discovery V8 stereomicroscope (Jena, Germany), with objective lenses of ×0.5 and ×1.0 and adjustable magnification of ×1.0–12.0), a high-speed camera (Photron Fastcam-Mini ×100 (San Diego, CA, USA), with a shooting speed ranging from 250 fps to 1000 fps), a back pressure valve (Coretest DBPR-5, Atlanta, GA, USA, with a maximum back pressure of 70 MPa and an accuracy of ±0.01 MPa), and an image acquisition system (NI system, Austin, TX, USA).

### 2.3. Experimental Process

Connect the experimental equipment according to the process shown in Figure 2 to simulate the seepage process of foamy oil in porous media. Through pressure reduction, the gas components in the foamy oil are precipitated in the form of bubbles, and the growth process of the bubbles is observed.

① Experimental preparation. The temperature of the thermostatic chamber is stabilized at 50 °C; the back pressure valve is set at 3.0 MPa; the foamy oil prepared in the intermediate container is at 3.0 MPa. Adjust the microscope, light source, and image acquisition device to achieve a state where clear images can be collected.

② Evacuate the glass model. Adjust the confining pressure of the visual high-pressure model to 1.0 MPa; turn on the vacuum pump to evacuate, and keep the vacuum gauge at −0.1 MPa for a stable period of no less than 1 h; then turn off the vacuum pump.

③ Saturate the degassed crude oil. In order to prevent gas precipitation due to a large pressure difference when saturating the foamy oil, the degassed crude oil is saturated first before saturating the foamy oil. Due to the effect of vacuum, the degassed crude oil flows into the glass model. When the pressure is balanced, the degassed crude oil is then saturated at a rate of 0.01 mL/min. Monitor the inlet pressure of the glass model. When the pressure approaches the confining pressure, increase the confining pressure and maintain a pressure difference of 1.0 ± 0.2 MPa. When the inlet pressure of the glass model reaches 2.0 MPa and the confining pressure is 3.0 ± 0.2 MPa, stop the saturation process.

④ Saturate the foamy oil. Slowly open the valve. As the pressure of the foamy oil is high, the foamy oil flows into the glass model. Although there is a tendency for bubbles to precipitate, the pressure process is very short and the gas will not precipitate. After the pressure is balanced, continue to saturate the foamy oil at a rate of 0.01 mL/min; maintain the confining pressure at 4.0 ± 0.2 MPa. When foamy oil is produced at the outlet and the form and flow rate of the produced foamy oil become stable, stop the saturation process.

⑤ Process of the foamy oil phase change experiment. Adjust the microscope and the clarity of the imaging again. For example, use the objective lens ×1.0 and observe at a magnification of 2 times. The image acquisition speed of the high-speed camera is set at 500 fps. Slowly adjust the back pressure valve and reduce the pressure from 3.0 MPa to 2.9 MPa within a period of no less than 10 min. During the adjustment process, carefully observe the image monitoring system, purposefully select phenomena such as bubble formation and growth, and record and store them using the high-speed camera. Similarly, the pressure reduction speed can be adjusted to achieve step-by-step pressure reduction and observation.

### 2.4. Numerical Simulation Method

#### 2.4.1. Molecular Dynamics Simulation Method

**Figure 3:**
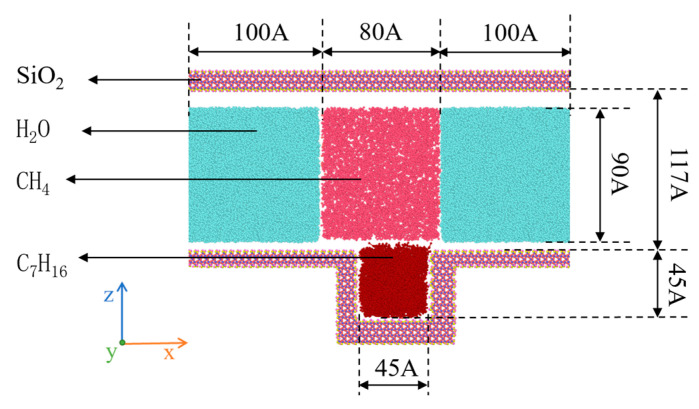
Fluid atom model of the SiO_2_ channel.

Molecular simulations were conducted using Material Studio to construct the model. The modeling process consists of two parts: the creation of the geometric model and the configuration of molecular distribution within the model. As shown in Figure 3, the overall model measures 29.4 × 5.4 × 19.2 nm^3^. The experimental glass etching model in the article utilized quartz glass, which contains SiO_2_, so a SiO_2_ unit cell was employed in the molecular dynamics simulations to better replicate the conditions. Fixed rigid walls were constructed in the z-direction, with the upper wall being flat and the lower wall featuring blind-end grooves filled with heptane molecules to make the diffusion phenomenon more apparent. Following the flow patterns of macroscopic fluids, periodic boundaries were set along the x and y directions, while non-periodic boundaries were applied in the z direction. The fluid can flow along the X and Y planes, while the Z direction has fixed boundaries. Periodic boundaries allow the fluid to exit from one side and simultaneously enter from the opposite side, whereas non-periodic boundaries restrict the fluid from entering the opposite side upon exiting. The temperature and pressure were set to 50 °C and 30 MPa, respectively, with 326 heptane molecules, 26,902 water molecules, and a methane density of 0.19 g/cm^3^, comprising 2300 methane molecules. Water molecules were used to replace other components in the oil phase to clearly illustrate the molecular interaction process between methane and heptane. The model was simulated using LAMMPS software (3 Mar 2020). The CVFF force field was used to construct the silicon dioxide substrate and heptane molecular layer, while the OPLS force field was applied to the aqueous and gas-phase regions. This combination ensures the accuracy of interfacial chemical reactions while optimizing the computational precision of solvation effects.

#### 2.4.2. Macro-Fluid Simulation Method

The volume change and migration process of bubbles are the main dynamic behaviors of bubbles when gas and liquid systems coexist in two phases. Generally, numerical and theoretical analyses of their morphological changes and migration behaviors are carried out through the continuity equation, momentum equation, and interface advancement equation.

For the calculation of multiphase flow, the continuity equation is usually used to track the gas–liquid interface and determine the position of the interface by calculating the volume proportion of each phase. If *q* is used to represent the gas phase or the liquid phase, the continuity equation for this phase is shown as follows:

(1) $$\frac{\partial \alpha_{q}}{\partial t}+u\cdot \nabla \alpha_{q}=\frac{S_{\alpha_{q}}}{\rho_{q}}$$

The right side of Equation (1) is the source term of the equation. Under normal circumstances, its magnitude is zero. In addition, in the initial state, the volume proportions of the gas and liquid phases do not need to be obtained through Equation (1), but through the following constraint relationships:

(2) $$\sum \alpha_{q}=1$$

It can be concluded from the constraint relationship (2) that for the mixed phase in each infinitesimal unit, the value of its overall physical property parameters is obtained by weighted averaging of each component dissolved in the liquid phase according to their respective volume proportions. It can thus be concluded that for a system with n-phase components, the weighted average density (weighted according to volume proportions) of any infinitesimal unit can be written as:

(3) $$\rho =\sum \rho_{q}\alpha_{q}$$

Since the bubble motion studied in this paper belongs to gas–liquid two-phase flow, the relevant physical property parameters of the gas phase and the liquid phase are now distinguished by subscript 1 and subscript 2 respectively. According to the constraint relationship of Equation (3), the weighted average density of any infinitesimal unit at this time is written as:

(4) $$\rho =\rho_{1}\alpha_{1}+\rho_{2}\alpha_{2}$$

Similarly, expressions for other parameters in the system can be obtained, and they will not be listed one by one here.

In the VOF (Volume of Fluid) algorithm, the momentum equations of the gas phase and the liquid phase are solved within the same calculation region. That is to say, in the process of solving the velocity field for the gas phase and the liquid phase, the same momentum equation is used for the solution. However, the physical property parameters in the equation, such as ρ (density) and µ (viscosity), are reflected by the volume-proportion-weighted average physical property parameters, and their magnitudes vary at different spatial positions. The momentum equation is as follows:

(5) $$\rho (\frac{\partial u}{\partial t}+u\cdot \nabla u)=-\nabla p+\nabla S+F_{s}+G$$

In Equation (5): S—Viscous shear tension S = µ(∇u + ∇Tu); Fs—Surface tension; G—Gravity term, G = ρg.

For the motion of bubbles, the gas–liquid interface is a free surface, and the physical property parameters of the fluids on both sides of it differ greatly, with differences spanning multiple orders of magnitude numerically. Therefore, it is very difficult to calculate the exchange of momentum and energy of the fluids on both sides, and it is also very hard to capture the precise position of the free surface. This poses high requirements for numerical calculations. Generally speaking, accurately simulating the two-phase flow field must meet the following several conditions: ① conservation of fluid mass; ② high-resolution interface; ③ accurate calculation of surface tension; ④ adaptability to a large ratio of physical property parameters of the two phases.

The VOF (Volume of Fluid) method is used to simulate two-phase flow through interface reconstruction, and the transport of physical quantities at the interface can adopt the Eulerian format or the Lagrangian convection format. Since this method is based on interface reconstruction, it can handle well the gas–liquid flow problems with large-deformation and complex interfaces, such as the problem of bubble coalescence. Moreover, by using the same set of equations to calculate the physical quantities related to the flow field, it can ensure mass conservation well, and the storage requirement during the calculation process is also relatively low. However, at the same time, when the VOF method uses the interface reconstruction method to calculate the gas–liquid two phases, there are problems such as discontinuity and intermittence in the reconstructed interface. Especially in three-dimensional cases, it is very difficult to obtain calculation results with high-order accuracy. We use the VOF method to capture the gas–liquid two-phase interface and use Youngs’ PLIC 3D algorithm (Piecewise Linear Interface Calculation) to reconstruct and transport the interface. We set the volume fraction of the fluid inside the interface as α = 0, the volume fraction of the fluid outside the interface as α = 1, and at the interface, α ranges from 0 to 1. Therefore, the transport equation of the interface is:

(6) $$\frac{\partial \alpha }{\partial t}+(u\cdot \nabla )\alpha =0$$

And for each computational grid, the physical property parameters at the center point of the grid, such as density and dynamic viscosity coefficient, can be obtained by volume-weighted averaging of each phase, that is, calculated through Equation (4).

There is surface tension on the gas-liquid interface. In the actual process of calculating the momentum equation, we use the Continuous Surface Force (CSF) model. The basic idea of this model is to equate the surface tension to a volume force source term and to equate the gas–liquid interface to a very thin transition zone in the flow field; the huge differences in the physical property parameters of the gas and liquid phases are smoothed out in this region. Through such processing, when calculating the gas–liquid two-phase flow, the same set of equations is used to solve for the two fluids. The expression for its surface tension is as follows:

(7) $$F_{s}=\sigma \kappa \delta_{s}n$$

(8) $$\kappa =-\nabla \cdot n=-\nabla \cdot (\nabla \alpha /\left|\nabla \alpha \right|)$$

**Figure 4:**
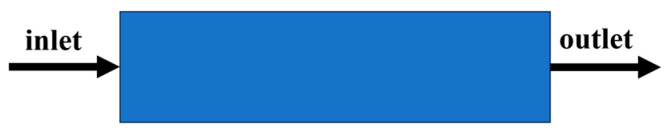
Schematic diagram of the geometric model.

To simulate the effects of gas component content, crude oil viscosity, and interfacial tension on bubble growth velocity, a straight-flow pipeline model, as shown in Figure 4, was designed. This model avoids the influence of pressure differences caused by pore structures on bubble morphology changes. The geometric dimensions of the two-dimensional model are 0.6 × 2.5 mm, with a structured square grid comprising 135,000 cells. The outlet pressure is set to 3.0 MPa, consistent with the experimental conditions, and the pressure difference between the inlet and outlet is 0.1 MPa, with the remaining boundaries being walls. The grid edge length is 3.3 μm, enabling the identification of bubbles with a diameter of 10 μm. Since the bubbles observed in the experiments are on the order of hundreds of micrometers, this grid resolution is sufficient for the requirements of this study.

Analyze the growth rate of bubbles through the change in the area of bubbles during the migration process. Calculate the area of bubbles according to the grid size and the number of grids. Use the ratio of the increased area to the migration time to represent the growth rate. The area of a single grid is 1.5 × 10^−6^ μm^2^.

## 3. Results and Discussion

### 3.1. The Variation Law of the Bubble Growth Rate in the Experiment

**Figure 5:**
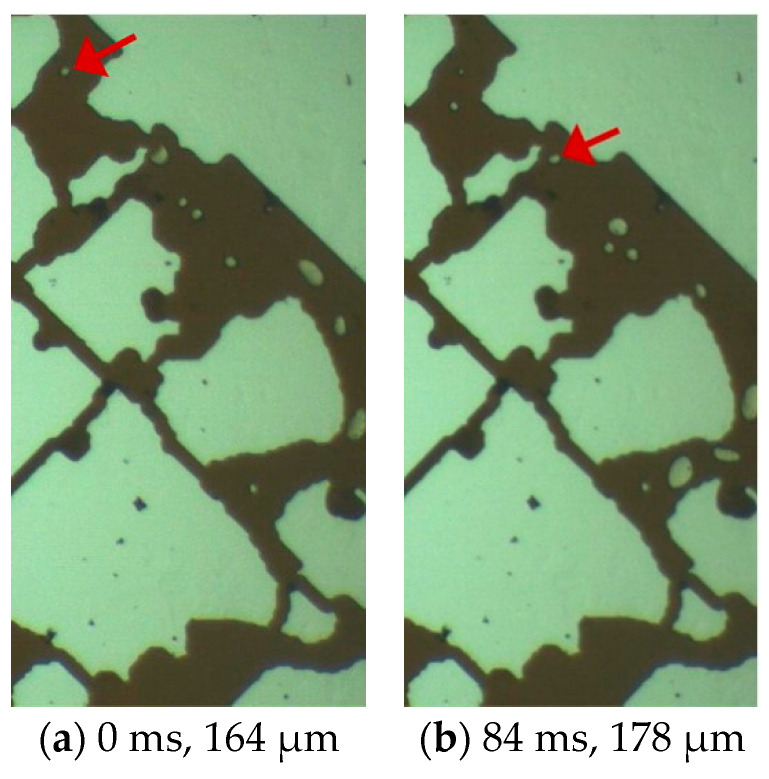

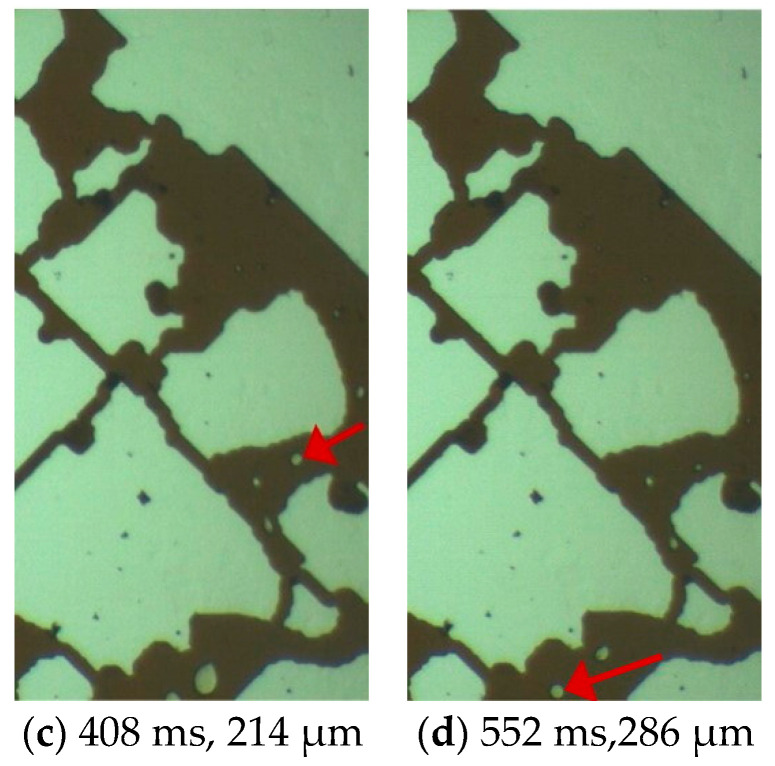
The growth process of bubbles during the migration process. The arrow in the figure points to the target bubble in the experimental analysis section.

Unlike a series of phenomena in conventional crude oil, such as rapid gas precipitation, rapid bubble growth and coalescence, the growth rate of bubbles in the depressurization experiment is relatively slow, and they grow while migrating within the pores. The bubbles indicated in Figure 5 are analyzed. In Figure 5a, the bubble diameter is 164 μm, which is much larger than the depth of the pores with a diameter of 20 μm, and the bubble is in a flattened state. The bubble shown in Figure 5a passes through a narrow throat and migrates to the position shown in Figure 5b in 84 ms, with the bubble diameter increasing to 178 μm. During the process from (a) to (b), the growth rate of the bubble is 0.17 μm/ms. At 408 ms, the bubble migrates to the position shown in Figure 5c, with the bubble diameter increasing to 214 μm. During the process from (b) to (c), the growth rate of the bubble is 0.11 μm/ms. During this process, the fluid enters large pores from small pores. According to the relationship between the flow velocity and the diameter in pipe flow, under the condition of a certain flow rate, the square of the pipe diameter is inversely proportional to the flow velocity. That is, when the pore diameter suddenly increases, the fluid will suddenly slow down. The change in the flow velocity causes the bubble to undergo elastic deformation under the action of inertia. The bubble diameter in Figure 5d is 286 μm. During the process from (c) to (d), the growth rate of the bubble is 0.50 μm/ms. The position of the bubble in Figure 5d is the limit position of the observation area. Based on the growth rate of the bubble at this moment, it can be judged that the bubble is still growing for a period of time after this moment.

**Figure 6:**
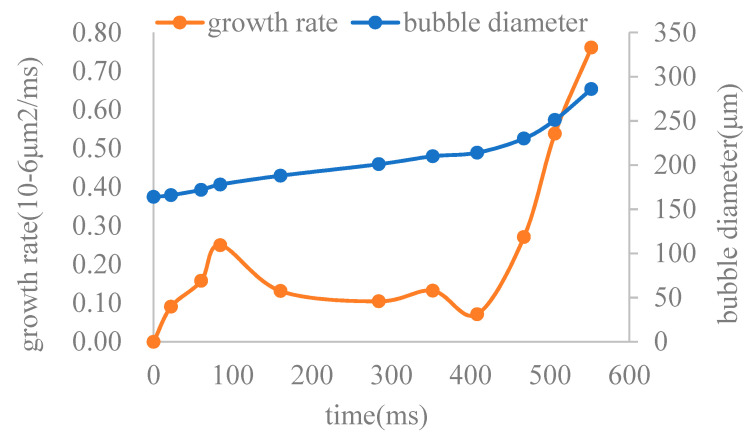
Bubble growth rate curve.

By tracking the bubbles in the observation area of Figure 5, it can be found that as the flow pressure difference decreases, the bubbles exhibit the phenomenon of simultaneous migration and growth. The growth rates of the bubbles in Figure 5 during various time periods are calculated, and the resulting regular curve is shown in Figure 6. According to the growth rate curve, the bubble growth can be divided into three stages: fast in the early stage, slowing down in the middle stage, and accelerating in the later stage. Based on the PV relationship, the bubble diameter is inversely proportional to the cube root of the pressure. As the migration distance increases, the growth rate of the bubbles gradually accelerates. However, according to the growth pattern in Figure 6, it indicates that the growth of bubbles in foamy oil is clearly not only affected by the pressure difference, and the main influencing factors and the degree of their influence are different in different growth stages.

The precipitation process of dissolved gas in conventional crude oil is continuous. Bubbles grow rapidly until they become stable, and the migration distance can be neglected. However, the bubble growth process in foamy oil presents three stages, and the bubble growth occurs simultaneously with the migration process. This phenomenon is significantly different from the conventional gas precipitation process. It is speculated that the high content of heavy components in foamy oil leads to the stability of the interface between the oil phase and the gas phase, resulting in the difficulty of degassing of foamy oil and a slow phase change process. The viscosity of foamy oil is higher than that of conventional crude oil, and the increase in viscosity will also slow down the growth and migration speed of bubbles. With different initial bubble diameters, under the action of the same pressure difference, the growth rates of bubbles will also be different.

There is a gas diffusion phenomenon in foamy oil. However, considering that the gas diffusion coefficient is related to the ambient temperature and pressure and is a common phenomenon in all gas–oil two-phase fluids, it has no particularity in the process of bubble growth. Therefore, the bubble growth process takes into account the combined effects of these three factors: crude oil viscosity, component content, and initial bubble diameter. However, through experimental research, multiple sets of controlled experiments need to be carried out, and it is difficult to control the pressure difference in the pores inside the microscopic model. So, it is impossible to study and verify the simulation results through experimental means. In order to accurately study the influence of single-factor parameters on bubble growth, numerical methods are adopted for simulation and analysis. The effects of pressure depletion rate, temperature, and other factors on bubble growth in foam oil have been extensively studied by previous researchers. Building on these prior studies, we have focused on highlighting the influence of three key factors in this article: gas component content, viscosity, and interfacial tension.

### 3.2. The Interaction Process Between Methane Molecules and Alkanes

**Figure 7:**
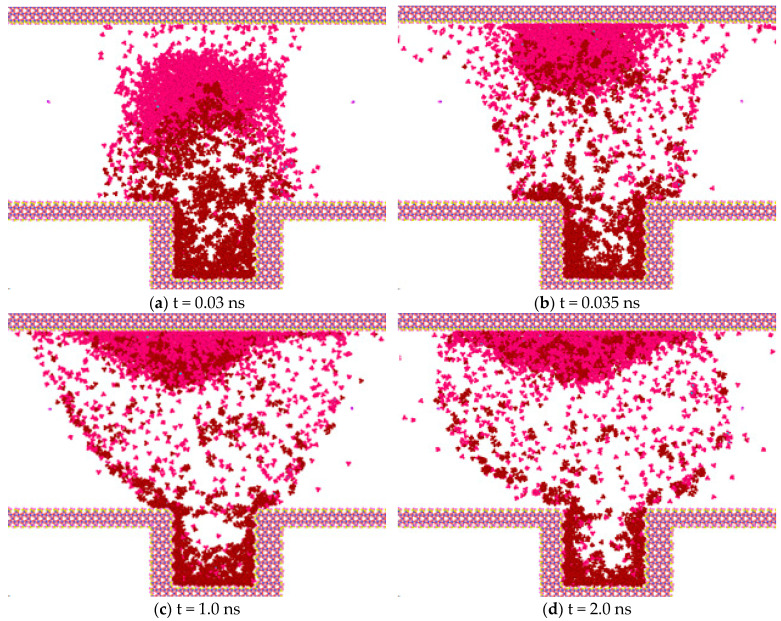
The interaction process of methane molecules and heptane molecules. The pink dots are methane molecules, and the dark red dots are heptane molecules.

In Figure 7, the pink dots are methane molecules, and the dark red dots are heptane molecules. For the convenience of analysis, when analyzing the diffusion process between methane molecules and heptane, other oil-phase molecules are hidden during the observation process so that the observation can be clearer.

Figure 7 shows the intrusion process of methane molecules and the separation process of heptane molecules at 50 °C and 30 MPa. Figure 7a presents the molecular diffusion state at 0.03 ns. Methane molecules, carrying some heptane molecules, start to diffuse in the z direction. Figure 7b shows the moment, at 0.035 ns, when methane molecules and a small number of heptane molecules are attached to the top wall surface of SiO_2_. In Figure 7c,d, methane molecules and heptane molecules continue to diffuse along the z direction and basically reach a dynamic equilibrium state of diffusion.

**Figure 8:**
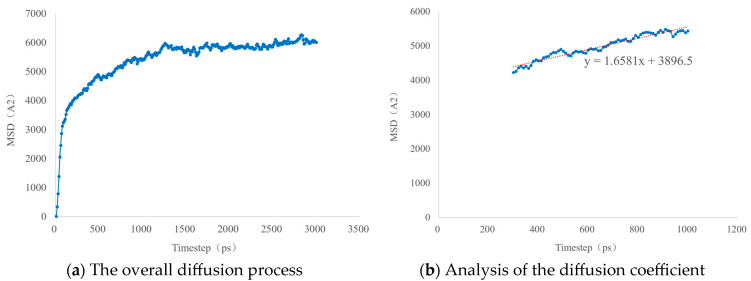
The diffusion degree of methane molecules in heptane molecules.

The diffusion process is plotted as the curve shown in Figure 8. At the beginning of the simulation, the first 0.03 ns is the stage for the system to conduct the initial structural optimization. After the optimization, the molecular diffusion speed increases. Around 1 ns, the diffusion phenomenon begins to enter a dynamic equilibrium state. Therefore, the diffusion speed is analyzed through the process from 0.03 ns to 1 ns. The slope of this curve can reflect the diffusion coefficient. According to Equation (9), the calculated diffusion coefficient is 2.8 × 10^−5^ cm^2^/s.

(9) $$D=\frac{k}{60000}$$

In the formula: D represents the diffusion coefficient, with the unit of cm^2^/s; k represents the slope, and its value is 1.6581.

The article investigates bubbles from a molecular scale and obtains the diffusion coefficient parameter of gas-phase molecules of bubbles within the oil-phase molecules. The flow state of bubbles in the oil phase belongs to the macroscopic level, and the aforementioned molecular diffusion coefficient parameter is utilized in subsequent numerical simulations of the flow state.

Component analysis of the oil sample reveals that the composition of foam oil is highly complex, with a high content of heavy components and dissolved gas, which gives rise to its unique physical properties. In the simulation, the essence of this characteristic—namely, the presence of bubbles within the oil phase—was captured without overemphasizing the complexity of the composition. For simulation convenience, the model was simplified to methane gas and a single-component oil phase.

### 3.3. Factor of the Content of Gas Components

**Figure 9:**
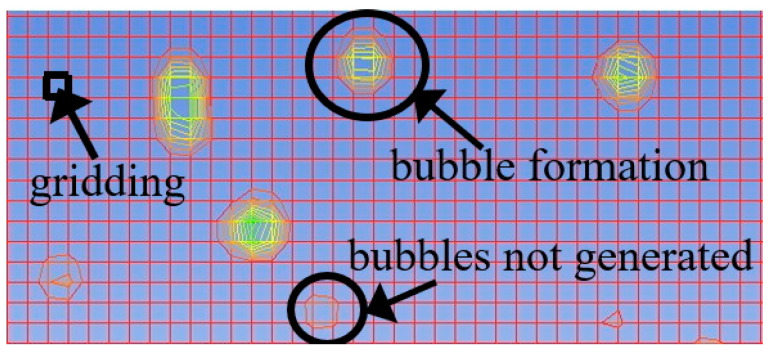
Illustration diagram of bubble generation size. Inside the model, red represents the oil phase, blue represents the gas phase, and other colors indicate the gas-liquid boundary.

The gas components in foamy oil affect the bubble growth process, while the heavy components have a greater impact on parameters such as viscosity. The lower the content of gas components is, the higher the corresponding content of heavy components will be. During the simulation, CH_4_ gas is taken as the representative of gas components, and the simulation results are analyzed based on the differences in CH_4_ content. The content of CH_4_ in the foamy oil prepared in the experiment is 12%. Therefore, numerical models with CH_4_ contents of 1%, 5%, 12%, 16%, and 20% in the fluid domain are established. When analyzing the bubble growth rate, the prerequisite is that the bubbles have already been generated, so a criterion for bubble generation is set. That is, under the current grid drawing conditions, if the number of clear bubble boundary layers that can be seen when magnified is greater than or equal to 5, it is considered that the bubbles have been generated, as shown in Figure 9. This paper focuses on the growth process of bubbles and does not consider the generation process of bubbles. The grid size affects the calculation accuracy of the bubble generation process, but it does not affect the growth process of bubbles.

**Figure 10:**
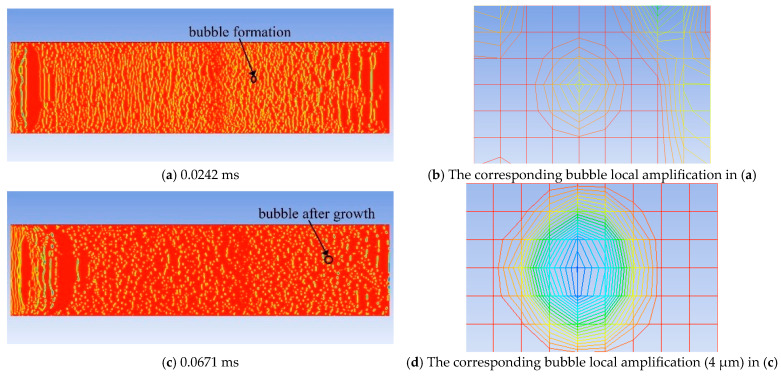
The bubble growth process when the CH_4_ content is 12% (@12 mPa·s). Inside the model, red represents the oil phase, blue represents the gas phase, and other colors indicate the gas-liquid boundary.

Figure 10 takes the simulation result with a CH_4_ content of 12% as an example to illustrate the growth rate of bubbles. In the figure, the red represents the oil phase, the blue represents the CH_4_ gas phase, and the color in the middle is the bubble boundary. The bubbles have already formed in Figure 10a, and magnified in Figure 10b. After 0.0671 ms, the bubbles grow, as shown in Figure 10c, and magnified Figure 10d. During this process, the growth rate of the bubbles is 8.7 × 10^−5^ μm^2^/ms.

**Figure 11:**
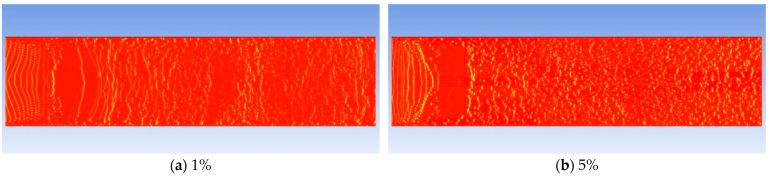

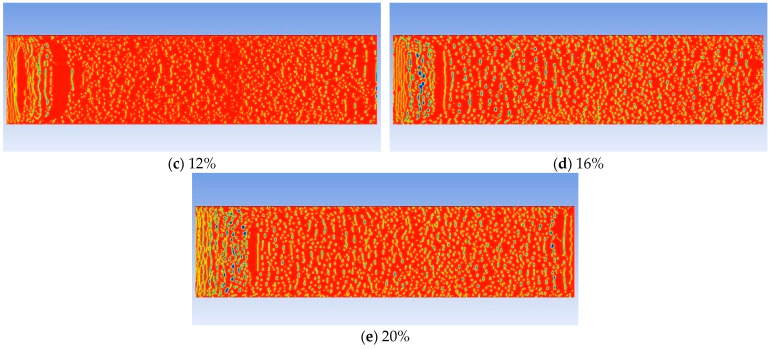
Comparative analysis of the bubble generation effects with different CH_4_ contents at the same time (@12 mPa·s). Inside the model, red represents the oil phase, blue represents the gas phase, and other colors indicate the gas-liquid boundary.

**Figure 12:**
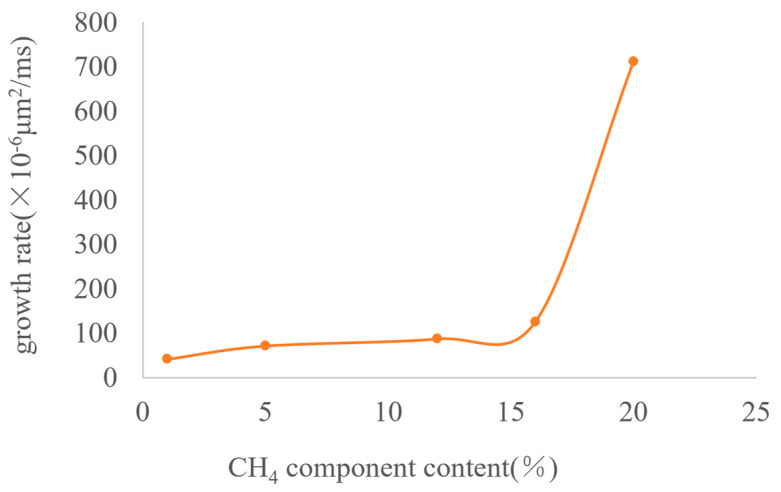
The curve of bubble growth rate at different CH_4_ contents.

As the content of gas components increases, the diameter of the generated bubbles becomes larger and the growth rate increases. Figure 11 shows images of bubbles after growth when the CH_4_ content is 1%, 5%, 12%, 16%, and 20%, respectively. The maximum diameters of the growing bubbles are 2 μm, 3 μm, 4 μm, 6 μm, and 13 μm, respectively. The curve of the bubble growth rate is plotted as shown in Figure 12. When the CH_4_ content is less than 15%, the variation range of the growth rate is relatively small. After exceeding 15%, the growth rate increases exponentially.

It can be seen from the above results that when exploited at the same depletion rate, the lower the content of gas components (that is, the higher the content of heavy components), the slower the bubble growth rate will be. This indicates that heavy components can play a key role in inhibiting bubble growth, prolonging the time for the oil reservoir to reach the bubble point pressure and extending the development stage of foamy oil flow. For the development of a specific oil reservoir, the component content of this oil reservoir is fixed, and it is impossible to extend the foamy flow time by controlling the component content. However, the foamy flow time in a specific oil reservoir can be predicted according to this curve so that the development strategy of the oil reservoir can be changed in a timely manner.

### 3.4. Viscosity Factor

**Figure 13:**
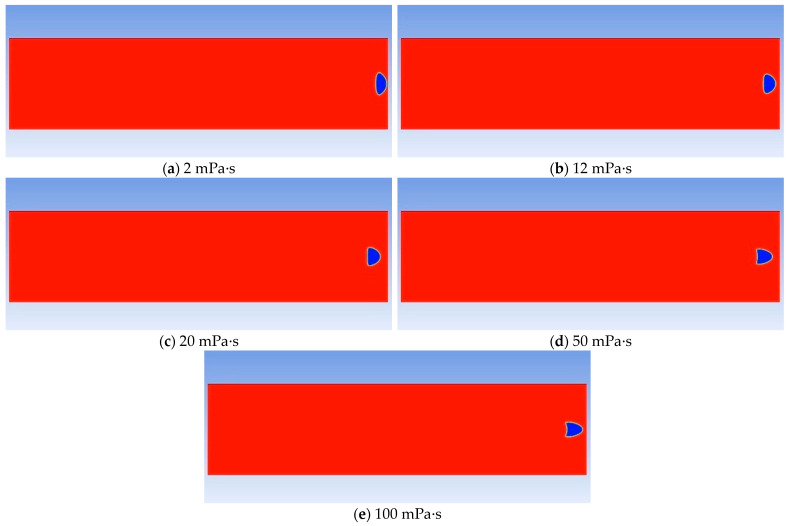
Comparison diagram of bubble shapes under different viscosities.

The growth and migration processes of bubbles are always affected by the viscous resistance of the oil phase. The greater the viscosity is, the greater the viscous force acting on the bubble boundary will be. Therefore, the degree of bubble deformation under different viscosities is different, and the growth rate is also slower. Taking the viscosity of the experimental foamy oil, which is 12 mPa·s, as the standard, a total of five groups of numerical models with viscosities of 2 mPa·s, 12 mPa·s, 20 mPa·s, 50 mPa·s, and 100 mPa·s were established to obtain the law of the effect of viscosity. In Figure 13, the shapes of bubbles are different under different viscosities of the oil phase. As the viscosity increases, the shape of bubbles gradually changes from an oval shape to a spherical cap shape and then to a crescent shape.

**Figure 14:**
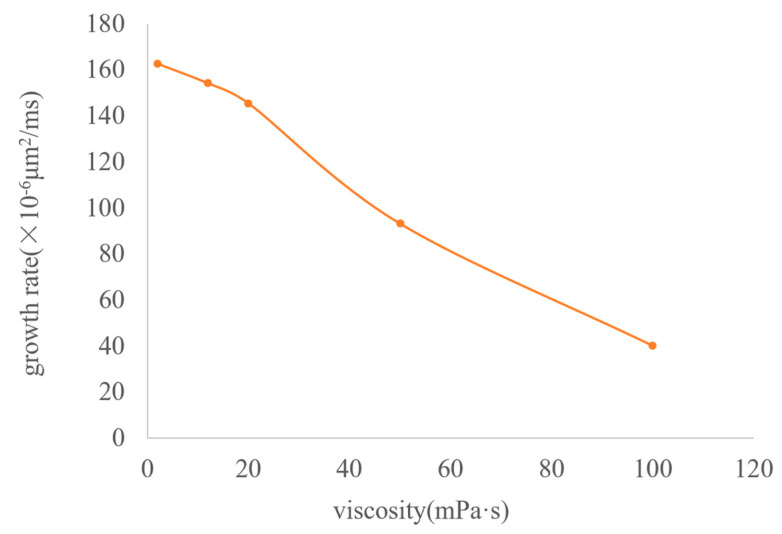
The curve of bubble growth rate under different viscosities.

The influence of viscosity on bubble growth is shown in the curve in Figure 14. As the viscosity increases, the growth rate of bubbles drops sharply, the migration speed becomes slower, and the time is prolonged. It can be seen from the above analysis that the viscosity of foamy oil is a key factor in inhibiting bubble expansion and can control the release speed of gas components. Reasonably controlling the viscosity of foamy oil is beneficial to extending the foamy flow stage.

### 3.5. Interfacial Tension Factor

Heavy components (such as asphaltenes and other high-molecular-weight organic substances) usually have relatively low interfacial activity, which may lead to the uneven formation of the interfacial film and instead increase the oil-water interfacial tension, especially at higher concentrations. These components may form a relatively thick film at the oil–water interface, increasing the interfacial tension and reducing the stability of the system.

**Figure 15:**
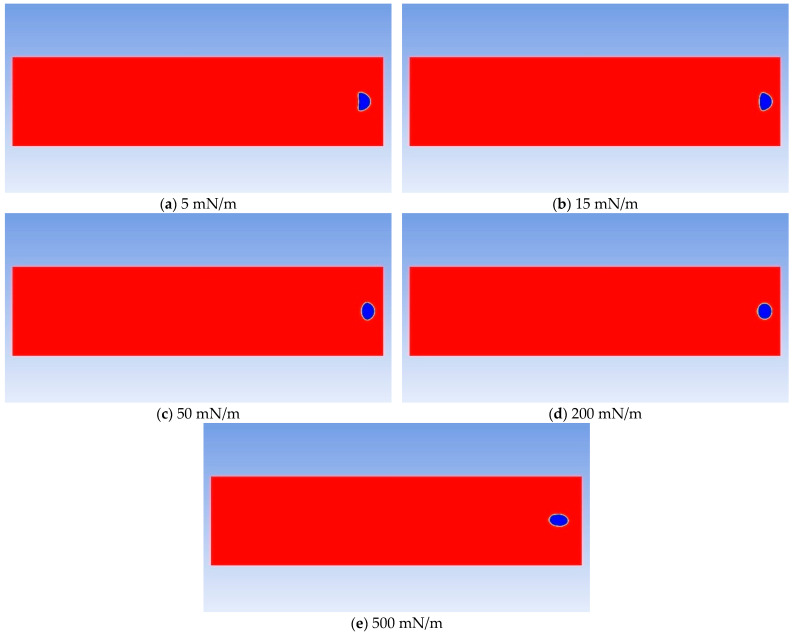
Comparison diagram of bubble shapes under different interfacial tensions.

The interfacial tension between CH_4_ and the oil phase is 15 mN/m. Based on 15 mN/m, a total of five groups of numerical models with interfacial tensions of 5 mN/m, 15 mN/m, 50 mN/m, 200 mN/m, and 500 mN/m were established. Figure 15 shows the simulation results of the influence of the magnitude of interfacial tension on the migration morphology of bubbles and the growth rate of bubbles. The simulation results indicate that as the interfacial tension increases, it is easier for the bubbles to maintain a spherical or ellipsoidal shape during the migration process.

**Figure 16:**
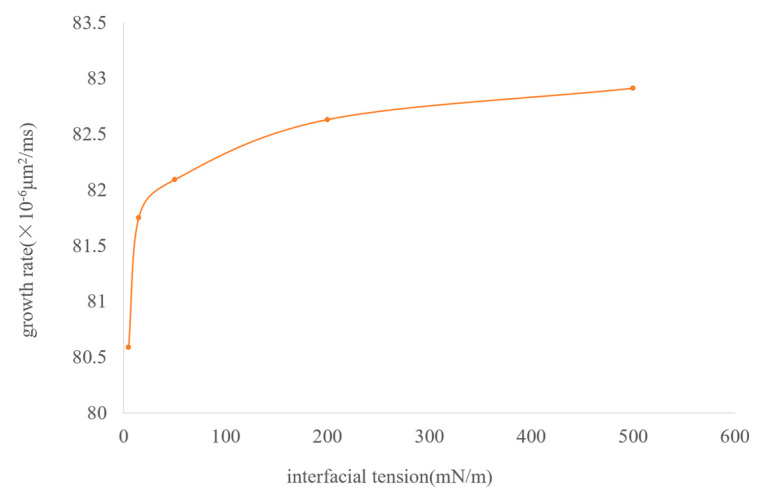
The curve of bubble growth rate under different interfacial tensions.

The influence of interfacial tension on bubble growth is shown in the curve in Figure 16. Generally speaking, when the interfacial tension is large, the growth rate of bubbles is also fast—in the range of 0.3 to 33.3 times the interfacial tension between CH_4_ and the oil phase—although the magnitude of the bubble growth rate does not change much and shows a certain regularity. It generally increases in a logarithmic form. As the interfacial tension increases, the degree of influence of the interfacial tension gradually weakens.

It can be known from the above simulation data that the variation coefficient of the bubble growth rate with the interfacial tension is close to 0. Therefore, from the perspective of oil reservoir exploitation, the influence of interfacial tension on the bubble growth rate can be neglected.

Based on the above single-factor simulation results, the bubble growth rate curve can be fitted in sections:

Effect of component content:

(10) $$f_{2}=\left\{\begin{array}{c} 18.416\ln\left(C\right)+41.716,\;C\leq 12\\ 2.9982e^{0.2621C},\;C>12 \end{array}\right.$$

Effect of viscosity:

(11) $$f_{3}=\left\{\begin{array}{c} -0.9541\mu +164.96,\;\mu \leq 20\\ -65.08\ln\left(\mu \right)+342.66,\;\mu >20 \end{array}\right.$$

Effect of interfacial tension:

(12) $$f_{4}=0.4666ln(\sigma )+80.152$$

In the formula: C represents the content of gas components, in %; μ represents the viscosity of the oil phase, in mPa·s; and σ represents the interfacial tension, in mN/m.

Based on the research findings of the aforementioned three influencing factors, the experimental data curves were fitted to derive the following equation:

(13) $$v=w_{1}f_{2}\left(C\right)+w_{2}f_{3}\left(\mu \right)+w_{3}f_{4}\left(\sigma \right)+\epsilon$$

In the equation: $w_{1}$, $w_{2}$, $w_{3}$ are the weighting factors for $f_{2}$, $f_{3}$, $f_{4}$, with values of 0.35, −0.5, and 0.15, respectively. ϵ represents the error term.

The ranking of the weights is ∣$w_{2}$∣ > $w_{1}\;$ > $w_{3}$, indicating that viscosity has the greatest influence, followed by gas content, while interfacial tension has the least impact.

In summary, based on the research results, during the extraction of foam oil, the recovery efficiency can be effectively enhanced by: ① Supplementing the gas component content in the formation through gas injection. ② Injecting surfactants to improve the stability of the foam fluid (by reducing interfacial tension and increasing fluid viscosity).

## 4. Conclusions

Based on the pressure-drop experiments on foamy oil, the growth process of the bubbles precipitated in the foamy oil was analyzed. Combining molecular simulation and Fluent numerical simulation methods, a quantitative analysis was conducted on factors such as the initial bubble diameter size, the component content of foamy oil, and the viscosity of foamy oil, and the following understandings were formed:

(1) The experiment found an important phenomenon that the growth of bubbles mostly occurs during the migration process. Under the condition of equal pressure difference reduction, the growth rate of bubbles presents a three-stage characteristic of being fast in the initial stage, slowing down in the middle stage, and accelerating in the later stage. It is analyzed that this is related to the different intensities of the roles played by complex factors in each stage. The experiment shows that after the bubble diameter reaches 200 μm, the growth rate increases exponentially. Therefore, during the development process, the bubble diameter of the precipitated bubbles should be controlled. A smaller bubble diameter is beneficial to improving the fluidity of foamy oil, while an overly large bubble diameter is likely to lead to an excessively fast growth rate of gas and reduce the utilization efficiency of gas energy.
(2) The numerical model shows that as the content of gas components in foamy oil increases, both the generation and growth rates of bubbles become larger. When the methane content is greater than 15%, the growth rate increases in an exponential trend. Combined with the slowly declining development characteristics of foamy oil, it is analyzed that the special fluid characteristics of foamy oil, which enable gas components to be slowly precipitated, are the key to its long-term development. It has been verified that the high content of heavy components in foamy oil leads to the stability of the interface between the oil phase and the gas phase, which is the main reason why foamy oil is not easy to degas and undergoes phase change slowly.
(3) The simulation results of the influence of viscosity on the bubble growth rate show that there is a logarithmic relationship between viscosity and growth rate. The greater the viscosity is, the slower the growth rate will be. It can be known from these simulation results that the viscosity of foamy oil is a key factor in inhibiting bubble expansion. It can control the release rate of gas components, enabling foamy oil to exhibit the characteristics of a long development time and a high recovery degree in depletion-type development.
(4) The interfacial tension has little impact on the bubble growth rate, and the influence of this factor can be neglected during the process of oil reservoir exploitation.

The experimental phenomena and the analysis of the numerical simulation results of the growth rate have explored the reasons for the slow growth of bubbles in foamy oil. The research results on the three influencing factors, namely the content of gas components, viscosity, and interfacial tension, have provided the exploitation ideas for controlling the pressure drop rate. Meanwhile, the research results on the content of gas components and viscosity factors can predict the exploitation status of foamy oil reservoirs and provide theoretical guidance for the timely adjustment of development strategies.

## Figures and Tables

**Table 1:** Analysis of degassed crude oil components.

Component	Components for the Preparation of Simulated Oil	Weight Ratio %
H_2_S, CO_2_, N_2_	/	0.00
C1~C9	/	0.00
C10~C19	C10	17.05
C20~C29	C20	23.95
C30+	C30	59.00
Total	C10, C20, C30	100.00

**Table 2:** Natural gas composition table.

Component	Weight Ratio %
CH_4_	85
C2~C3	10
C4	5

## Data Availability

The raw/processed data required to reproduce these findings cannot be shared at this time, as these data also form part of an ongoing study.
